# The pressing need for FDA regulation of tattoo ink

**DOI:** 10.1093/jlb/lsae014

**Published:** 2024-07-08

**Authors:** Lisa A Verity, Ana Santos Rutschman, Michael S Sinha

**Affiliations:** Barklage, Brett & Hamill, P.C., St. Charles, MO, USA; Villanova University Charles Widger School of Law, Villanova, PA, USA; Center for Health Law Studies, Saint Louis University School of Law, St. Louis, MO, USA

Approximately 30 per cent of Americans—including 40 per cent of adults aged 18–34—have at least one tattoo.^1^ Tattoo ink, however, poses several health-related concerns.^2^ Heavy metals found in inks—including cadmium, lead, mercury, antimony, beryllium, and arsenic—have been shown to cause cancer, degenerative brain diseases, and cardiovascular and endocrine abnormalities.^3^ The FDA notes that the risks involved with the application of tattoos include injection and removal problems, allergic reactions, granulomas, keloid formation, and MRI complications.^1^

Tattoo needles typically go 1–2 mm into the skin to permanently suspend the ink in the matrix of the dermis. The immune system tries to clear the ink from the body; unable to do so, the ink remains stored in white blood cells until cell death, when the ink is again released into the dermal matrix and the process starts over. White blood cells can also carry ink throughout the body—tattoo ink has been found in lymph nodes and in some cases has mimicked malignancy and made staging of cancer more difficult.^4^

In this Essay, we argue that greater regulatory oversight is urgently needed. Standardization and consistency in the manufacturing and use of tattoo ink would protect consumers from exposure to unknown ingredients that may cause allergic reactions, infection, or other serious illnesses.

## I. FDA CLASSIFICATION OF TATTOO INK: COSMETIC, DRUG, OR COLOR ADDITIVE?

The FDA currently classifies tattoos as cosmetics and relies on voluntary public reporting of adverse events, which leads to vast amounts of underreporting. The FDA also has regulatory authority over color additives used in cosmetics but declines to exercise authority over color additives in tattoo ink ‘because of other competing public health priorities and a previous lack of evidence of safety problems specifically associated with these pigments.’^4^ We show here that tattoo ink fits within the legal definition of ‘color additive,’ for which the Federal Food, Drug, and Cosmetic Act (FDCA) imposes a more stringent regulatory regime.

The FDCA defines a ‘cosmetic’ as a product ‘introduced into or otherwise applied to the human body or any part thereof for…beautifying, promoting attractiveness, or altering the appearance.’ (21 U.S.C. § 321(i)) But the pigments in tattoo ink also qualify as a ‘color additive,’ defined in the law as a ‘dye, pigment, or other substance [that] … when added or applied to … the human body or any part thereof, is capable … of imparting color thereto.’ (21 USC § 321(t)) Under this provision, ‘color’ includes black, white, and different shades of gray.

The FDA is required to assess whether color additives are safe for their intended purpose. As a rule, color additives used in cosmetics must be approved by the FDA, and certain color additives must be batch-certified. Throughout this process, the FDA makes a determination that the use of a given color additive for a specific purpose is likely safe.

Although the law gives the FDA the ability to regulate tattoo ink more stringently, to date, the agency has not approved any color additives for injection.

## II. ADULTERATION, MISBRANDING, AND SAFETY

The FDCA prohibits the use or introduction into commerce of adulterated or misbranded cosmetics. Adulteration in a cosmetic occurs when: it may cause injury to the user because it contains a potentially harmful substance; it contains filth; it contains a non-permitted color additive; or it is manufactured in unsanitary conditions. Misbranded cosmetics are those that have false or misleading labeling; do not list required information; or have a misleading container.

The regulatory regime currently applicable to tattoo ink is weak. The voluntary cosmetic registration system (VCRP) makes determinations of adulteration and misbranding of cosmetics very difficult. The VCRP does not apply to systems or items that are for professional use only, and product filings are voluntary, submitted using best estimates about the products. Less than 10 per cent of all cosmetic businesses in the USA are registered through the VCRP—voluntary reporting is ripe for underreporting.^5^

Consumer safety would be advanced if tattoo ink manufacturers were required to register with the FDA, subject their products to regular inspections and testing, and routinely submit safety data to the FDA. Registration and inspections lead to higher quality, consistent products while discouraging adulteration, and better data generated through standardized adverse event reporting would help identify risks associated with tattoo ink.

Given the impact of tattoo ink on human health and the FDA’s clear authority to regulate color additives in tattoo ink, closer scrutiny of ink components is warranted. Such scrutiny would also facilitate the gathering of data on, and the study of, heavy metals, plastics, and other potentially harmful additives in tattoo ink. This knowledge would in turn lead to a more robust understanding of the impact of these components on human health, thus providing FDA with a better framework to fine-tune future regulation of tattoo ink.

## III. RECLASSIFYING TATTOO INK

Recently, the FDA issued a Draft Guidance focused on the preparation, packing, and holding of tattoo ink to prevent contamination.^6^ We believe the agency needs to go further. Many of these problems could be solved with a new regulatory scheme for tattoo ink. An option would be to include tattoo ink into a class of ‘injectable cosmetics.’ This class would encompass cosmetics that are injected into or under the skin that are permanent, semi-permanent, or temporary in nature. As proposed, the class would include products like Botox and Juvéderm, but also tattoo ink.

Injectable cosmetics that contain drugs or color additives tend to be riskier and more long-lasting than regular cosmetics—some of these products are already classified as both a drug or medical device and a cosmetic. Tattoo ink should be reclassified to allow the FDA to extend regulatory authority over the ink’s ingredients, instill labeling requirements, and monitor its use more closely.

The growing popularity of tattoos, risks associated with adverse events, allergic reactions, and inadvertent injection of heavy metals all suggest a need for tighter regulation. In 2022, Congress passed the Modernization of Cosmetics Regulation Act (MoCRA),^7^ described by the FDA as ‘the most significant expansion of FDA’s authority to regulate cosmetics since the [FDCA] was passed in 1938.’^8^ MoCRA now requires cosmetic manufacturers to register facilities and list products with the agency to track product quality and enable inspection of facilities. MoCRA also mandates serious adverse event reporting to the FDA and allows for recalls if there is a reasonable probability that the product is adulterated or misbranded. Other important reforms include good manufacturing processes and product listing requirements.

Despite these important reforms within MoCRA, it remains unclear whether these requirements will be extended to tattoo ink, as recent guidance implores consumers and healthcare providers—and not manufacturers—to report adverse reactions from potentially contaminated tattoo inks.^6^ Ideally, the FDA should apply provisions of MOCRA to tattoo inks as injectable cosmetics, but at a minimum, should exercise its regulatory authority over color additives to establish guidelines for additives allowed in tattoo ink.

## Footnotes

1 U.S. FDA, *Think Before You Ink: Tattoo Safety*, https://www.fda.gov/consumers/consumer-updates/think-you-ink-tattoo-safety (accessed Feb. 15, 2024).

2 B. M. Kiszla, C. A. Elmets & T. T. Mayo, *Quantitative Analysis of Restricted Metals and Metalloids in Tattoo Inks: A Systematic Review and Meta-Analysis*, 313 Chemosphere 137291 (2023).

3 S. Negi, L. Bala, S. Shukla & D. Chopra, *Tattoo Inks are Toxicological Risks to Human Health: A Systematic Review of their Ingredients, Fate Inside Skin, Toxicity due to Polycyclic Aromatic Hydrocarbons, Primary Aromatic Amines, Metals, and Overview of Regulatory Frameworks*, 38 Toxicol. Ind. Health 417, 434 (2022).

4 T. Paul Litton & S. Vijay Ghate, *Tattoo Pigment Mimicking Axillary Lymph Node Calcifications on Mammography*, 15 Radiol. CaseRep. 1194, 1196 (2020).

5 E. P. Sites & A. E. Woodruff, *Tattoos: Regulatory Loopholes and Who’s Responsible, Public Health Post* (2021), https://www.publichealthpost.org/research/tattoos-regulatory-loopholes-and-whos-responsible/ (accessed Feb. 15, 2024).

6 U.S. FDA, *FDA Issues Draft Guidance on Tattoo Inks*, https://www.fda.gov/cosmetics/cosmetics-news-events/fda-issues-draft-guidance-tattoo-inks (accessed Apr. 21, 2024).

7 Modernization of Cosmetic Regulation Act (MoCRA), Pub. L. 117–328, 2023.

8 U.S. FDA, *Regustration & Listing of Cosmetic Product Facilities and Products*, https://www.fda.gov/cosmetics/registration-listing-cosmetic-product-facilities-and-products (accessed Apr. 21, 2024).

